# Genome-wide analysis of Dongxiang wild rice (*Oryza rufipogon* Griff.) to investigate lost/acquired genes during rice domestication

**DOI:** 10.1186/s12870-016-0788-2

**Published:** 2016-04-26

**Authors:** Fantao Zhang, Tao Xu, Linyong Mao, Shuangyong Yan, Xiwen Chen, Zhenfeng Wu, Rui Chen, Xiangdong Luo, Jiankun Xie, Shan Gao

**Affiliations:** College of Life Sciences, Jiangxi Normal University, Nanchang, Jiangxi 330022 P. R. China; College of Animal Science and Veterinary Medicine, Shanxi Agricultural University, Taigu, Shanxi 030801 P. R. China; Department of Biochemistry and Molecular Biology, College of Medicine, Howard University, Washington DC, WA 20059 USA; Tianjin Institute of Crop Research, Tianjin Academy of Agricultural Sciences, Tianjin, 300381 P. R. China; College of Life Sciences, Nankai University, Tianjin, 300071 P. R. China; School of Mathematical Sciences, Nankai University, Tianjin, 300071 P. R. China; Tianjin Institute of Agricultural Quality Standard and Testing Technology, Tianjin Academy of Agricultural Sciences, Tianjin, 300381 P. R. China

**Keywords:** Dongxiang wild rice, Whole genome sequencing, Transcriptome, Comparative genomics analysis, Structural variation

## Abstract

**Background:**

It is widely accepted that cultivated rice (*Oryza sativa* L.) was domesticated from common wild rice (*Oryza rufipogon* Griff.). Compared to other studies which concentrate on rice origin, this study is to genetically elucidate the substantially phenotypic and physiological changes from wild rice to cultivated rice at the whole genome level.

**Results:**

Instead of comparing two assembled genomes, this study directly compared the Dongxiang wild rice (DXWR) Illumina sequencing reads with the Nipponbare (*O. sativa*) complete genome without assembly of the DXWR genome. Based on the results from the comparative genomics analysis, structural variations (SVs) between DXWR and Nipponbare were determined to locate deleted genes which could have been acquired by Nipponbare during rice domestication. To overcome the limit of the SV detection, the DXWR transcriptome was also sequenced and compared with the Nipponbare transcriptome to discover the genes which could have been lost in DXWR during domestication. Both 1591 Nipponbare-acquired genes and 206 DXWR-lost transcripts were further analyzed using annotations from multiple sources. The NGS data are available in the NCBI SRA database with ID SRP070627.

**Conclusions:**

These results help better understanding the domestication from wild rice to cultivated rice at the whole genome level and provide a genomic data resource for rice genetic research or breeding. One finding confirmed transposable elements contribute greatly to the genome evolution from wild rice to cultivated rice. Another finding suggested the photophosphorylation and oxidative phosphorylation system in cultivated rice could have adapted to environmental changes simultaneously during domestication.

**Electronic supplementary material:**

The online version of this article (doi:10.1186/s12870-016-0788-2) contains supplementary material, which is available to authorized users.

## Background

Cultivated rice (*Oryza sativa* L.), as one of the most important agricultural crops, supplies the main dietary source for more than half of the world’s population [1]. Although it is well accepted that cultivated rice was domesticated from common wild rice (*Oryza rufipogon* Griff.) thousands of years ago [2], the origin and domestication process of cultivated rice have been debated for decades through different studies [3–5]. Until recently, it was revealed that *O. sativa* L. ssp*. japonica* had been first domesticated from a specific population of *O. rufipogon* around the middle area of the Pearl river in southern China, and that *O. sativa* L. ssp*. indica* had been subsequently developed from crosses between *japonica* and local wild rice as the initial cultivars spread into Southeast and South Asia [6]. Along with a multitude of studies on rice origin, further work is needed to genetically elucidate the substantially phenotypic and physiological changes from wild rice to cultivated rice at the whole genome level.

This study conducted comparative genomics analysis between *O. sativa* L. spp. *japonica* var. Nipponbare and Dongxiang wild rice (DXWR), a Chinese common wild rice (*O. rufipogon*). DXWR was firstly discovered in Dongxiang county, Jiangxi province of China in 1978 [7], which was considered as the most northern one (28°14’N) of the regions where common wild rice populations had been discovered around the world. During the past three decades, DXWR has been well investigated as a precious genetic resource for cultivated rice improvement or fundamental research on genetic diversity [8, 9], heterosis [10], cytoplasmic male sterility [11], fertility restoration [12], biomass [13], high yield [14–16], and resistance to biotic and abiotic stress [17–21].

To perform the comparative genomics analysis, we sequenced the whole genome of DXWR using Next-Generation Sequencing (NGS) technologies. In this study, the strategy of the comparative genomics analysis was to directly compare the DXWR NGS reads with the Nipponbare complete genome without assembly of the DXWR genome. This strategy avoided the highly time-consuming work and a substantial number of errors resulted from the *de novo* assembly of the DXWR genome using the NGS short reads. The essential work in this comparative strategy was using the software SVDetect and the pipeline SVFilter to detect structure variations (SVs), which are being increasingly appreciated for their roles as a cause for phenotypic variations [22–24]. Using the detected deletions (one important type of SVs), we located genes which could have been acquired by Nipponbare during rice domestication. To overcome the limit of the SV detection, the DXWR transcriptome was also sequenced and compared with the Nipponbare transcriptome to discover the genes which could have been lost in DXWR during domestication. Both Nipponbare-acquired genes and DXWR-lost transcripts were further analyzed using annotations from multiple sources (e.g. QTLs for traits and KEGG pathways) to reach two research goals: 1) to help better understanding the domestication from wild rice to cultivated rice at the whole genome level; 2) to provide a genomic data resource for rice genetic research or breeding.

## Results and discussion

### Whole-genome sequencing of Dongxiang wild rice

The sequencing of the Dongxiang wild rice (DXWR) genome produced a total of 282,383,842 paired-end 90 bp cleaned reads (25.4 Gb data) using Illumina sequencing technology, covering 68-fold of the reference genome (*O. sativa* L. spp. *japonica* var. Nipponbare) with the size 373,245,519 bp. The high depth of this Next-Generation Sequencing (NGS) data satisfied the requirement for the reliable detection of structure variations (SVs). Then, we mapped all the cleaned reads to nine complete rice genomes (Methods). Using single-end alignment of forward-sequenced reads, the rate of mapped reads against the total reads reached 74.19, 56.98, 47.11, 36.29, 32.02, 29.30, 19.76, 4.20 and 1.18 % for *O. sativa* L. spp. *japonica* var. Nipponbare, *O. sativa* L. spp. *indica*, *O. nivara*, *O. glaberrima*, *O. barthii*, *O. glumaepatula*, *O. meridionalis*, *O. punctata* and *O. brachyantha*, respectively. Nipponbare reached the highest mapped rate probably due to two reasons. The first reason is that *O. sativa* is considered to have been domesticated from Chinese common wild rice [2]. The second reason is that the Nipponbare genome was sequenced using the clone-by-clone approach with Sanger sequencing technology and is ranked as the best assembled and annotated one out of all rice genomes. Therefore, we used the Nipponbare genome as reference to detect SVs.

### Structural variations between Dongxiang wild rice and Nipponbare genome

We used the software SVDetect to detect SVs between DXWR paired-end reads and the Nipponbare genome without assembly of the DXWR genome. The basic theory of SVDetect is to use priori information from paired-ends such as order, orientation and insert size of pairs (500 bp in this study) to classify mapped reads into normally and anomalously paired-end reads. Removing normally mapped paired-end reads, SVDetect uses anomalously mapped paired-end reads to produce SVs. Since SVDetect produces a large number of false positive SVs, we developed a pipeline named SVFilter to reduce the false positives. SVFilter uses five independent programs (ratiofilter, gapfilter, SNVfilter, coveragefilter and depthfilter) to successively filter out false positives (Methods). In this study, SVDetect produced 13,767 potential SVs and the ratiofilter largely reduced the SV number to 3946 (28.66 % of 13,767). Then, the gapfilter, SNVfilter, coveragefilter and depthfilter narrowed down the SV number to 3945, 3524, 2570 and 2539 (Additional file 1), respectively.

**Fig. 1:**
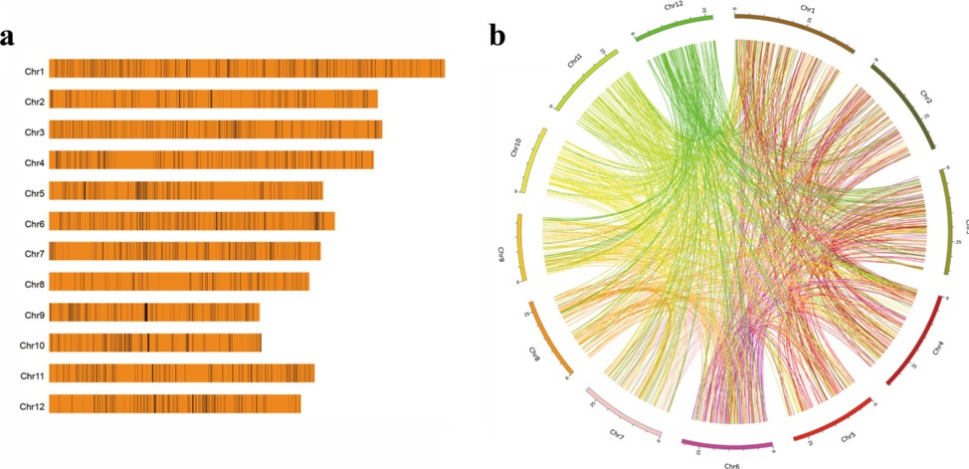
Distribution of deletions and translocations on Nipponbare genome. **a** Totally 1568 deletions were determined between the Dongxiang wild rice and Nipponbare genome. **b** Totally 437 translocations and 423 inverted translocations were plotted on 12 rice chromosomes using Nipponbare as reference

**Fig. 2:**
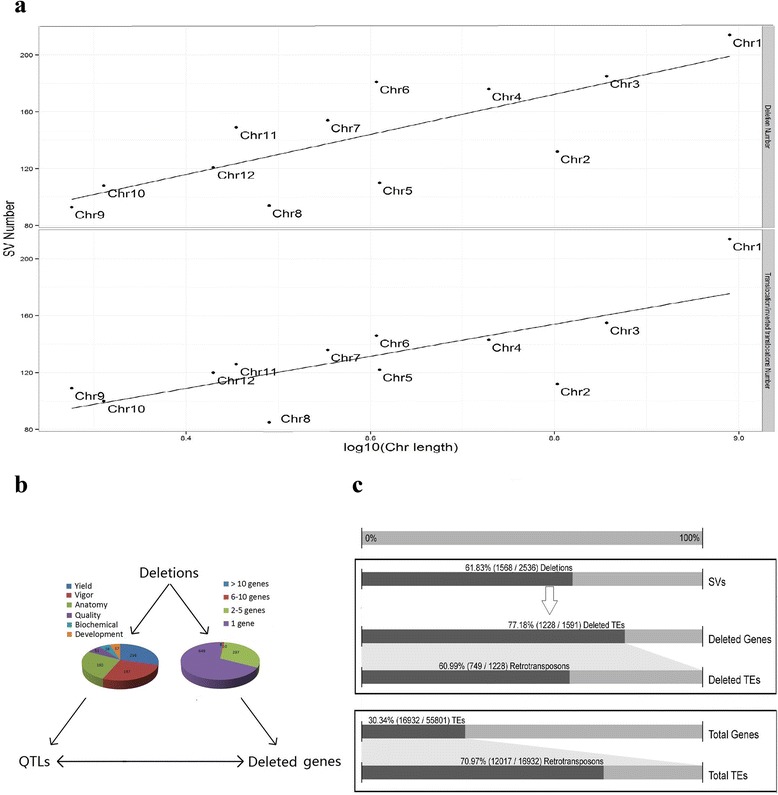
Further analysis of 1568 deletions. **a** Relationship between the deletion & translocation/inverted translocation number and the chromosome length on a logarithmic scale. **b** Relationship between deletions, deleted genes and QTLs. **c** Transposable elements and retrotransposons comparison between the Nipponbare genes in the genome and Nipponbare-acquired genes during domestication

After removing the larger deletions which overlapped the smaller inside deletions, 1568 out of 1571 deletions were left for further analysis. Finally, 2536 SVs were determined to include 1568 deletions, 437 translocations, 423 inverted translocations, 88 inversions, six inverted duplications, three inverted fragment inversions, one fragment insertion and 10 undefined SVs. Among eight types of SVs, the deletions contribute to 61.83 % (1568/2536) of the total SVs (Fig. 1a), followed by translocations/inverted translocations accounting for 33.91 % (860/2536) of the total SVs (Fig. 1b). Generally speaking, the deletion number and the translocation/inverted translocation number has a linear relationship with the chromosome length on a logarithmic scale, respectively (Fig. 2a). All chromosomes of the Nipponbare genome contain the deletions and translocations/inverted translocations in the same pattern with the exception of chromosome 9. Moreover, chromosome 1 contains more deletions and translocations/inverted translocations than the other 11 chromosomes.

### Discovery of Nipponbare-acquired genes during rice domestication

The SV detection and filtering process determined 1568 deletions. These deleted regions could contain genes which were hypothesized to have been acquired by Nipponbare during rice domestication. Mapping the 1568 deletions to rice genes in the Nipponbare genome, 61.47 % (964/1568) of the total deletions were associated to 1591 genes (Additional file 2). Among 964 deletions, 67.32 % (649/964) contain only one gene. The top eight deletions containing more than ten genes were mapped to the genomic region from 10790002 bp to 10920963 bp on chromosome 10 (Chr10:10790002–10920963), Chr9:10545593–10732410, Chr8:9242722–9284595, Chr2:14239399–14298646, Chr6:23563294–23592857, Chr6:29241098–29330521, Chr2:17681691–17753826 and Chr11:11280103–11340819 (Fig. 2b). Among 1591 Nipponbare-acquired genes, 77.18 % (1228/1591) are transposable elements (TEs), 60.99 % (749/1228) of which are retrotransposons (Fig. 2c). In the total 55,801 Nipponbare genes, 30.34 % (16,932/55,801) are TEs, 70.97 % (12,017/16,932) of which are retrotransposons. More than two-fold (77.18 vs. 30.34 %) difference of TE percentages is consistent with the previous finding that TEs contribute greatly to the genome evolution from wild rice to cultivated rice [25].

Quantitative Trait Loci (QTLs) link particular regions on the genome to the agronomic traits. In rice, numerous QTLs for important agronomic traits have been identified and included in the Gramene QTL database (Methods). Mapping the deletions to the annotated rice QTLs in the Nipponbare genome, 731 QTLs located in 539 unique regions on the Nipponbare genome were associated to 937 deletions (Additional file 3). The phenotypes of these 731 QTLs belong to six trait categories. They are yield (216 of 731), vigor (197), anatomy (192), quality (51), biochemical (38) and development (37) (Fig. 2b). Combining the results from the previous steps, we constructed the relationship between QTLs and genes in the same regions affected by the deletions (Additional file 4). Finally, the relationship between 937 deletions, 1547 deleted genes and 731 QTLs was constructed for further studies (Fig. 2b). Using this relationship information, agronomic traits, QTLs and associated genes were summarized to help better understanding the domestication from wild rice to cultivated rice. During this domestication process, cultivated rice could have acquired genes involving plant height, spikelet number, panicle number, leaf senescence and panicle length on the top five traits, followed by biomass yield, seedling vigor, leaf width, tiller number and various other traits (Table 1).

**Table 1:** QTLs for traits and associated genes

Trait	QTL number	Gene number
Plant height	86	1338
Spikelet number	71	1019
Panicle number	39	460
Leaf senescence	37	669
Panicle length	37	772
Biomass yield	33	442
Seedling vigor	32	416
Leaf width	25	456
Tiller number	25	434
1000-seed weight	22	504
Root number	21	459
Root thickness	20	285
Seed dormancy	20	299
Chlorophyll content	17	271
Leaf length	17	324
Spikelet density	16	472
Root length	15	237
Seed number	15	545
Anther length	9	257
Culm thickness	9	336
Awn length	8	168
Culm length	8	253
Seed length	8	138
Grain yield	7	71
Mesocotyl length	7	67
Yield	7	264
Carbohydrate content	6	124
Carbon content	6	184
Grain length	6	113
Grain number	6	199
Panicle weight	6	296
Primary branch	6	70
Root activity	6	48
Flour color	5	78
Leaf area	5	84
Seed width	5	110
100-seed weight	4	148
Germination speed	4	35
Grain weight	4	45
Head rice	4	91
Amylose content	3	16
Consistency viscosity	3	78
Gelatinization temperature	3	43
Grain shattering	3	82
Grain width	3	21
Ratooning ability	3	150
Rubisco content	3	161
Secondary branch	3	69
Spikelet weight	3	138
Chlorophyll ratio	2	25
Flower number	2	12
Gel consistency	2	10
H2O2 content	2	18
Leaf height	2	82
Setback	2	12
Breakdown viscosity	1	5
Groat percentage	1	51
Leaf perimeter	1	5
Photosynthetic ability	1	21
Protein content	1	4
Root volume	1	27
Seed density	1	22
Seed weight	1	15

The records were sorted by the QTL number. Trait used annotations from the Gramene database v40. Additional file 4 records the relationship between QTLs and rice genes in the same region on the reference chromosome

### Discovery of DXWR-lost genes using the DXWR transcriptome

In this study, we only used 500 bp paired-end reads from the DXWR genome for SV detection. This single library size limited the size of detected insertions on the DXWR genome to not more than 300 bp. Then, we could not detect large insertions (>300 bp) to locate DXWR-lost genes during rice domestication. Therefore, we sequenced and assembled the DXWR transcriptome to discover DXWR-lost genes. The total RNA was extracted from seedling leaves and seedling roots to construct two separate RNA-seq libraries, which were sequenced on the Illumina HiSeq 2000 system. After data cleaning and quality control, a total of 86,340,332 paired-end 100 bp raw reads (8.6 Gbp data) were processed to 85,813,832 cleaned reads, with the Q20 percentage of 99.3 %. These cleaned reads were *de novo* assembled into the DXWR transcriptome, filtering out contigs shorter than 200 bp.

**Fig. 3:**
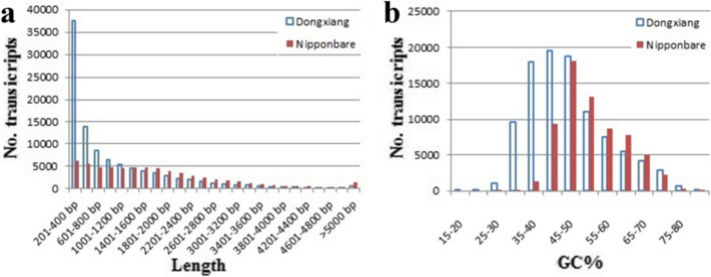
Dongxiang wild rice and Nipponbare transcriptome. Dongxiang and Nipponbare represents the Dongxiang wild rice and Nipponbare transcripts longer than 200 bp. The Dongxiang transcriptome could contain transcripts from chloroplast or mitochondria. **a** Length distribution. **b** GC-content distribution

The DXWR transcriptome contains 70,747 genes and 99,092 transcripts with the average length 968 bp and the N50 length 1655 bp, while the Nipponbare transcriptome contains 55,204 genes and 65,556 transcripts with the average length 1722 bp and the N50 length 2295 bp, filtering out the transcripts shorter than 200 bp. The length distribution of the assembled DXWR transcriptome was compared with the length distribution of the Nipponbare transcriptome (Fig. 3a). From Fig. 3a, it can be seen the number of DXWR and Nipponbare transcripts decreases with the transcript length in a similar pattern, with an exception of transcripts shorter than 1000 bp. Particularly, DXWR has more short transcripts (<500 bp) than Nipponbare does. This difference is mainly due to the incomplete assembly of these DXWR transcripts using the NGS short reads. Although 45,737 (46.16 % of 99,092) DXWR transcripts have lengths less than 500 bp, 53,355 (53.84 % of 99,092) transcripts with lengths at least 500 bp still provide abundant information for further studies. In addition, Nipponbare has more high-GC transcripts than DXWR does (Fig. 3b). The previous study indicated that high-GC genes in rice and other cereals have greater bias and codon usage is skewed toward codons that are preferred in highly expressed genes [26].

Using the program blastx, 60,247 (60.8 % of 99,092) transcripts were annotated by the functional description of their top 20 similar sequences (hits), if they existed, from the NCBI Non-Redundant Protein (NR) database (Additional file 5). Using the software blast2go, 35,596 (35.92 % of 99,092) transcripts were annotated by Gene Ontology (GO) slim terms for plants in three domains, molecular function, cellular component and biological process. By mapping GO slim terms to enzyme codes and the Kyoto Encyclopedia of Genes and Genomes (KEGG) database, 4548 (4.59 % of 99,092) transcripts were assigned to 137 KEGG pathways (Additional file 6). Using the program blastn, 99,092 DXWR transcripts were mapped to the Nipponbare transcriptome and then, 409 unmapped transcripts were mapped to the Nipponbare mitochondrial cDNA, chloroplast cDNA and nuclear genome sequences. Finally, 206 transcripts from 205 genes were identified as DXWR-lost transcripts during domestication.

### Further analysis of Nipponbare-acquired and DXWR-lost genes

**Fig. 4:**
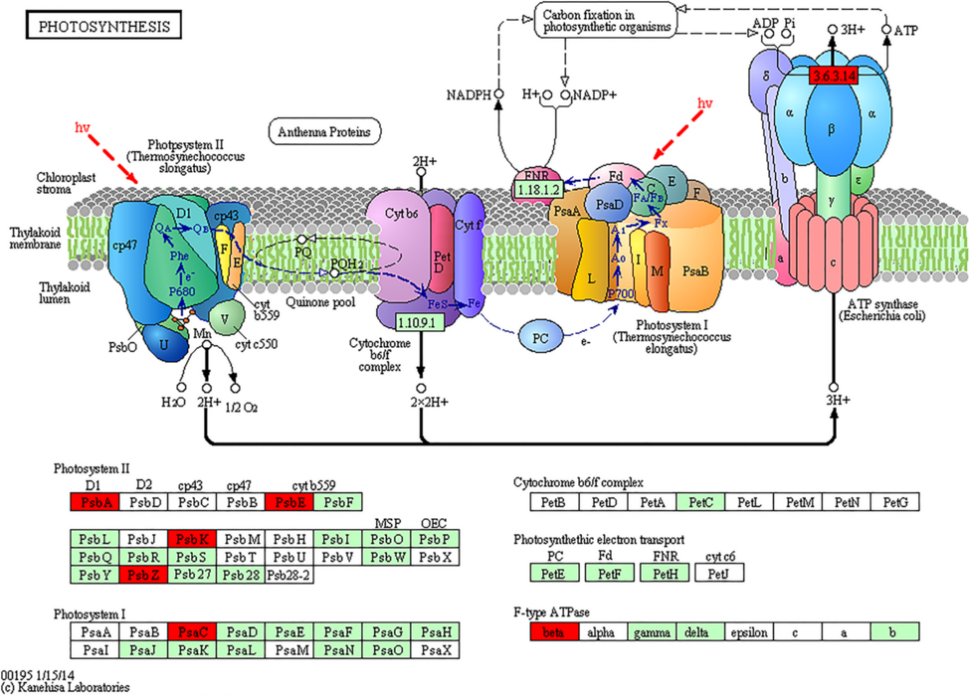
The photosynthesis pathway with Nipponbare-acquired genes. This figure (KEGG: map00195) was downloaded from the KEGG website with copy-right permission. Among 1591 Nipponbare-acquired genes, 13 photosynthesis related genes were mapped to six proteins (Additional file 7: Table S2). They are photosystem II PsbA, photosystem II PsbE, photosystem II PsbK, photosystem II PsbZ, photosystem I PsaC and F-type ATPase beta (*in red*)

**Fig. 5:**
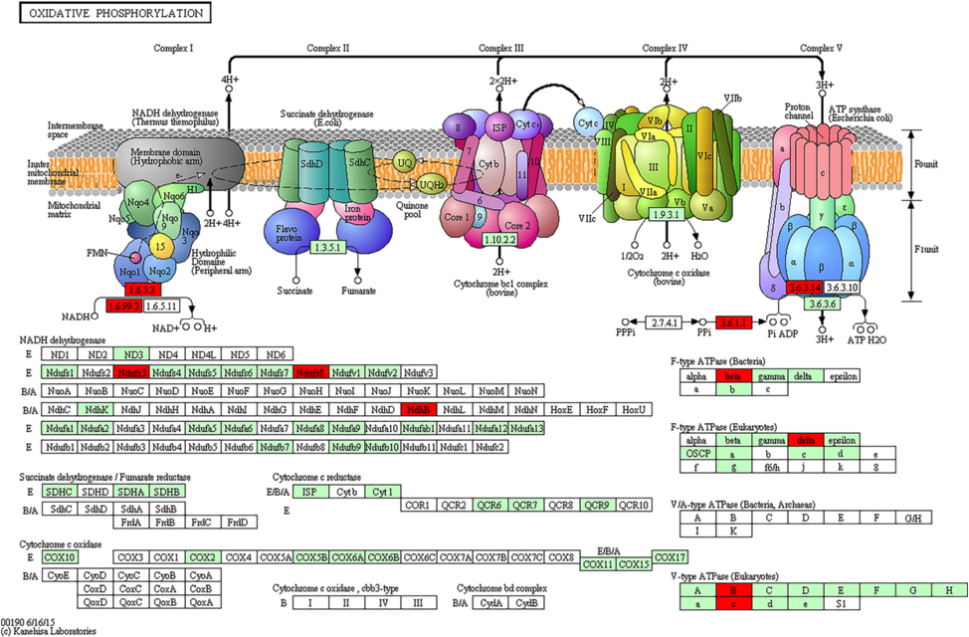
The oxidative phosphorylation pathway with Nipponbare-acquired genes. This figure (KEGG: map00190) was downloaded from the KEGG website with copy-right permission. Among 1591 Nipponbare-acquired genes, 11 oxidative phosphorylation related genes were mapped to eight proteins (Additional file 7: Table S3). They are NADH dehydrogenase E Ndufs3, NADH dehydrogenase E Ndufs8, NADH dehydrogenase B/A NdhB, F-type ATPase (Bacteria) beta, F-type ATPase (Eukaryotes) delta, V-type ATPase (Eukaryotes) B, V-type ATPase (Eukaryotes) c and inorganic pyrophosphatase [EC:3.6.1.1] (*in red*)

To further analyze 1591 Nipponbare-acquired genes, 1652 putative proteins encoded by them were mapped to KEGG pathways, known transcription factors (TFs) and protein kinases (PKs), respectively. The significant results included 13 genes involving the photosynthesis pathway (Fig. 4), 11 genes involving the oxidative phosphorylation pathway (Fig. 5), three putative proteins encoded by two genes (*LOC_Os08g33488* and *LOC_Os12g31748*) belonging to the transcription factor MIKC, three putative proteins encoded by three genes (*LOC_Os09g29540*, *LOC_Os09g29560* and *LOC_Os09g29584*) belonging to the wall associated kinase (WAK) or wall associated kinase-like kinase family, seven putative proteins encoded by the gene *LOC_Os01g20900* belonging to the receptor like cytoplasmic kinase VIII family and five putative proteins encoded by the gene *LOC_Os01g27020* belonging to the CDC2 like kinase family. Further analysis showed 18 of 1591 Nipponbare-acquired genes have only one copy in the genome. These 18 genes encode proteins from HNH endonuclease family, trehalose phosphatase, MYB family transcription factor, ubiquitin-protein ligase 1 and peptide transporter PTR2, *et al.* (Additional file 7). Further analysis of 206 DXWR-lost transcripts showed 18 of them had functional annotations and three of them were enriched in the oxidative phosphorylation pathway. These three transcripts encode three proteins, which are cytochrome b, cytochrome oxidase subunit I and NAD-dependent aldehyde dehydrogenase (Additional file 7).

MIKC as one type of MADS-box TFs has been implicated in several aspects of plant growth and development. Genome-wide identification and phylogenetic analyses of 75 MADS-box genes in rice showed: 1) the Nipponbare-acquired gene *LOC_Os08g33488* (*OsMADS23*) and *LOC_Os12g31748* (*OsMADS20*) was categorized into the AGL17-like and the SQUA-like group, respectively; 2) these two MADS-box genes were genetically far from other genes within their groups [27]. The WAK gene family plays important roles in cell expansion, pathogen resistance and heavy-metal stress tolerance in *Arabidopsis*. The OsWAK gene family containing 125 members expanded in the rice genomes due to lineage-specific expansion of the family in monocots [28]. The Nipponbare-acquired gene *LOC_Os09g29540* (*OsWAK82*), *LOC_Os09g29560* (*OsWAK83*) and *LOC_Os09g29584* (*OsWAK84*) was classified into the type of OsWAK-RLCK, OsWAK pseudogene and OsWAK-RLP [28], respectively.

In total, 13 Nipponbare-acquired genes were enriched in the photosynthesis pathway, 11 Nipponbare-acquired genes and three DXWR-lost transcripts were enriched in the oxidative phosphorylation pathway (Additional file 7). Both photophosphorylation and oxidative phosphorylation are potential ATP sources for the plasmalemma ATPase, which drives the phosphorylation reaction to releases energy. Previous studies showed these two energy systems cooperate to provide energy. One example is mitochondrial oxidative phosphorylation appears to serve an essential function for supplying the cytosol with ATP during photosynthesis [29]. Another example is photophosphorylation in the light and oxidative phosphorylation in the dark supply energy together for the opening of stomata on the epidermal peels of *Commelina communis* L. and *Vicia faba* L. [30]. Previous studies also showed that cultivated rice possesses higher photosynthetic efficiency than wild rice does to produce high yield [31, 32]. These findings together suggest the photophosphorylation and oxidative phosphorylation system in rice could have adapted to environmental changes simultaneously during domestication from wild rice to cultivated rice.

## Conclusions

In this study, we performed the whole genome sequencing of Dongxiang wild rice (DXWR), a Chinese common wild rice. Using the software SVDetect and the pipeline SVFilter, 2536 structural variations (SVs) were determined between DXWR and the reference genome Nipponbare. Out of 2536 SVs, 1568 deletions were used to locate 1591 deleted genes, which were hypothesized to have been acquired by Nipponbare during its domestication. To overcome the DNA library size limit, the DXWR transcriptome was sequenced and compared with the Nipponbare transcriptome to obtain 206 DXWR-lost transcripts during rice domestication. Further analysis of Nipponbare-acquired genes and DXWR-lost transcripts showed 13 Nipponbare-acquired genes were enriched in the photosynthesis pathway. In addition, 11 Nipponbare-acquired genes and three DXWR-lost transcripts were enriched in the oxidative phosphorylation pathway. The photophosphorylation and oxidative phosphorylation system in rice could have adapted to environmental changes simultaneously during domestication from wild rice to cultivated rice.

In this study, the relationship between 937 deletions, 1547 deleted genes and 731 QTLs was constructed to provide a valuable resource for further studies. Using this relationship information, agronomic traits, QTLs and associated genes were summarized to help better understand the substantially phenotypic and physiological changes from wild rice to cultivated rice at the whole genome level. This information can also be used to guide the future experiments for rice genetic research or breeding.

## Methods

### DNA and RNA sequencing of Dongxiang wild rice

Dongxiang wild rice (DXWR) is *ex situ* conserved in Jiangxi Academy of Agricultural Sciences, Nanchang, China (http://www.jxaas.com/index.html), and the seeds of DXWR are freely available for scientific research. In order to avoid the interference from extraneous pollen and reduce the heterozygosity, DXWR had been subjected to self-pollination by bagging cultivation for more than ten years. The seeds used in this study were acquired with a Material Transfer Agreement (MTA) from the Jiangxi Academy of Agricultural Sciences. Firstly, the seeds of DXWR were growing under natural conditions in a paddy field at the experimental station. Then, the DXWR seeds from the paddy field were planted in the growth chamber (28 ± 2 °C, 14 h/day and 10 h/night) to acquire seedlings for DNA and RNA extraction. The experimental research reported here complies with institutional, national, and international guidelines concerning plant genetic repositories.

The DNA was extracted from the seedling leaves and the total RNA was from seedling leaves and seedling roots at the four-leaf stage, separately. The DNA-seq library with the 500 bp insert size was constructed using Illumina TruSeq DNA Sample Prep Kit and sequenced using the 90 bp paired-end technology on the Illumina HiSeq 2000 system. Two non-strand-specific RNA-seq libraries with the 200 bp insert size were constructed using Illumina TruSeq RNA Sample Prep Kit and sequenced using the 100 bp paired-end technology on the Illumina HiSeq 2000 system. The cleaning and quality control of the DNA-seq and RNA-seq data were conducted using the pipeline Fastq_clean [33] that is optimized to clean the raw reads from Illumina platforms [34–41]. Low quality (< Q20) and adapter contained reads were removed from the DNA-seq data. As for the RNA-seq data processing, low quality (< Q20) nucleotides on both ends of the raw reads were trimmed and the trimmed reads which contain ambiguous nucleotides (“N”) at least two were removed. Then, adapter segments on the 3’ ends of the remaining reads were trimmed and short (<25 bp) trimmed reads were removed. Finally, the virus-like and rRNA-like RNA-seq reads were removed.

### Rice genomes, MSU annotation and QTL data

Nine complete genome data were downloaded from the Ensembl database v22 (ftp://ftp.ensemblgenomes.org/pub/release-22/plants). The sequences with annotation of the reference genome (*O. sativa* L. spp. *japonica* var. Nipponbare) in Ensembl v22 are identical to the data in IRGSP v1.0 and MSU_osalr v7.0 (MSU Rice Genome Annotation Project Release 7). The gene and protein annotation used MSU_osalr v7.0 (http://rice.plantbiology.msu.edu/). The rice QTL data were downloaded from the Gramene database v40 (http://www.gramene.org) [42]. After redundancy removal, the number of QTL records was narrowed down from 8216 to 5165.

### Software and programs

The software Trinity r20140717 [43] was used to assemble the DXWR transcriptome. The software bowtie v0.12.7 [44] was used to align the cleaned DNA reads to nine complete rice genomes allowing two mismatches. The software bwa v0.5.7-r1310 was used to align the cleaned DXWR DNA reads to the Nipponbare genome for SV detection. SV detection was conducted using the software SVDetect v0.8b [45]. The pipeline SVFilter was developed in Fei’s lab, Cornell University, which can be downloaded from http://bioinfo.bti.cornell.edu/tool/SVFilter/. This paper is required to be cited to use SVFilter. The parameters for bwa, SVDetect and SVFilter in command lines were provided in Additional file 7. Mapping deletions to genes or QTLs was conducted using in-house Perl programs. Genome graphs (e.g. Fig. 1) were plotted using the software Circos v0.66 [46]. Statistics and plotting were conducted using the software R v2.15.3 with the package ggplot2 [47].

### Availability of data and materials

The NGS data are available in the NCBI SRA database with ID SRP070627. All the supporting data are included as additional files.

### Ethics

Not applicable.

### Consent to publish

Not applicable.

## Additional files

Additional file 1: This file reports 2539 SVs (=links) detected between Dongxiang wild rice and Nipponbare as reference. This file is in the tab delimited format and can be opened using the software Excel. (TXT 1496 kb) Additional file 2: This file reports mapping the 1568 deletions to rice genes in the reference genome. This file is in the tab delimited format and can be opened using the software Excel. (TXT 268 kb) Additional file 3: This file reports mapping the 1568 deletions to QTLs. This file is in the tab delimited format and can be opened using the software Excel. (TXT 1015 kb) Additional file 4: This file records the relationship between QTLs and rice genes in the same region on the reference chromosome. This file is in the tab delimited format and can be opened using the software Excel. (TXT 2342 kb) Additional file 5: This file reports the functional annotation of 99,092 DXWR transcripts from the NCBI NR database using the software blast2go. This file is in the tab delimited format and can be opened using the software Excel. (TXT 12649 kb) Additional file 6: This file reports 137 KEGG pathways of 4548 DXWR transcripts. This file is in the tab delimited format and can be opened using the software Excel. (TXT 275 kb) Additional file 7:
**Table S1.** Eighteen of 1591 Nipponbare-acquired genes. **Table S2.** Thirteen Nipponbare-acquired genes involving the photosynthesis pathway. **Table S3.** Eleven Nipponbare-acquired genes involving the oxidative phosphorylation pathway. **Table S4.** Eighteen DXWR-lost transcripts during domestication. The last section recorded all the parameters used in bwa, SVDetect and SVFilter. (DOC 126 kb)

## Abbreviations

Chr: chromosome
DXWR: Dongxiang wild rice
GO: Gene Ontology
KEGG: Kyoto Encyclopedia of Genes and Genomes
NGS: Next-Generation Sequencing
NR: Non-Redundant Protein
PK: protein kinase
QTLs: Quantitative Trait Loci
SV: structural variation
TE: transposable element
TF: transcription factor
WAK: wall associated kinase
