# Supplementary material for: Genome-wide analysis of Dongxiang wild rice (Oryza rufipogon Griff.) to investigate lost/acquired genes during rice domestication
Source: BMC Plant Biol. 2016 Apr 26;16:103. doi: 10.1186/s12870-016-0788-2 (PMC4845489; doi:10.1186/s12870-016-0788-2)
Supplement: Additional file 7: — Table S1. Eighteen of 1591 Nipponbare-acquired genes. Table S2. Thirteen Nipponbare-acquired genes involving the photosynthesis pathway. Table S3. Eleven Nipponbare-acquired genes involving the oxidative phosphorylation pathway. Table S4. Eighteen DXWR-lost transcripts during domestication. The last section recorded all the parameters used in bwa, SVDetect and SVFilter. (DOC 126 kb) [file 12870_2016_788_MOESM7_ESM.doc]

**Table S1. Eighteen of 1,591 Nipponbare-acquired genes**

| **MSU ID** | **Chr** | **Start** | **End** | **Protein name** |
| --- | --- | --- | --- | --- |
| LOC_Os01g27020 | Chr1 | 15059422 | 15067554 | transposon protein, putative, unclassified, expressed |
| LOC_Os01g66480 | Chr1 | 38594450 | 38594743 | expressed protein |
| LOC_Os02g14510 | Chr2 | 7997539 | 7997865 | expressed protein |
| LOC_Os02g33470 | Chr2 | 19904790 | 19906960 | transposon protein, putative, Pong sub-class, expressed |
| LOC_Os04g40200 | Chr4 | 23919033 | 23920878 | HNH endonuclease family protein, putative, expressed |
| LOC_Os05g24684 | Chr5 | 14294663 | 14308828 | structural constituent of ribosome, putative, expressed |
| LOC_Os06g03230 | Chr6 | 1220784 | 1221326 | hypothetical protein |
| LOC_Os06g11840 | Chr6 | 6277593 | 6281865 | trehalose phosphatase, putative, expressed |
| LOC_Os06g21516 | Chr6 | 12423323 | 12425493 | transposon protein, putative, Pong sub-class, expressed |
| LOC_Os06g36870 | Chr6 | 21723220 | 21725390 | transposon protein, putative, Pong sub-class, expressed |
| LOC_Os07g40380 | Chr7 | 24210969 | 24212734 | expressed protein |
| LOC_Os08g33050 | Chr8 | 20528292 | 20535292 | MYB family transcription factor, putative, expressed |
| LOC_Os08g36179 | Chr8 | 22821989 | 22822849 | expressed protein |
| LOC_Os09g07900 | Chr9 | 4016486 | 4036019 | ubiquitin-protein ligase 1, putative, expressed |
| LOC_Os09g18440 | Chr9 | 11304720 | 11306890 | transposon protein, putative, Pong sub-class, expressed |
| LOC_Os10g02260 | Chr10 | 805663 | 809801 | peptide transporter PTR2, putative, expressed |
| LOC_Os10g42920 | Chr10 | 23141918 | 23142342 | conserved hypothetical protein |
| LOC_Os12g09410 | Chr12 | 4931702 | 4933198 | retrotransposon protein, putative, LINE subclass, expressed |

Further analysis showed 18 of 1,591 Nipponbare-acquired genes have only one copy in the genome.

**Table S2. Thirteen Nipponbare-acquired genes involving the photosynthesis pathway**

| **MSU ID** | **Chr** | **Start** | **End** | **Protein name** |
| --- | --- | --- | --- | --- |
| LOC_Os02g24634 | Chr2 | 14286575 | 14287636 | photosystem II PsbA protein |
| LOC_Os10g21192 | Chr10 | 10807276 | 10808337 | photosystem II PsbA protein |
| LOC_Os06g39728 | Chr6 | 23580417 | 23580668 | photosystem II PsbE protein |
| LOC_Os08g15322 | Chr8 | 9279906 | 9281314 | photosystem II PsbE protein |
| LOC_Os10g21298 | Chr10 | 10869191 | 10869442 | photosystem II PsbE protein |
| LOC_Os02g24642 | Chr2 | 14288946 | 14289590 | photosystem II PsbK protein |
| LOC_Os10g21198 | Chr10 | 10814227 | 10814412 | photosystem II PsbK protein |
| LOC_Os02g24628 | Chr2 | 14284410 | 14284598 | photosystem II PsbZ protein |
| LOC_Os10g21214 | Chr10 | 10819061 | 10819249 | photosystem II PsbZ protein |
| LOC_Os10g21406 | Chr10 | 10913946 | 10914191 | photosystem I PsaC protein |
| LOC_Os03g55874 | Chr3 | 31819698 | 31820959 | F-type ATPase beta |
| LOC_Os06g39740 | Chr6 | 23589443 | 23590939 | F-type ATPase beta |
| LOC_Os10g21266 | Chr10 | 10858911 | 10860407 | F-type ATPase beta |

Protein name is from the figure 4 in the manuscript.

**Table S3. Eleven Nipponbare-acquired genes**

**involving the oxidative phosphorylation pathway**

| **MSU ID** | **Chr** | **Start** | **End** | **Protein name** |
| --- | --- | --- | --- | --- |
| LOC_Os10g21258 | Chr10 | 10855090 | 10855569 | NADH dehydrogenase E Ndufs3 |
| LOC_Os10g21398 | Chr10 | 10911669 | 10912282 | NADH dehydrogenase E Ndufs8 |
| LOC_Os08g15248 | Chr8 | 9255763 | 9258007 | NADH dehydrogenase B/A NdhB |
| LOC_Os03g55874 | Chr3 | 31819698 | 31820959 | F-type ATPase (Bacteria) beta |
| LOC_Os06g39740 | Chr6 | 23589443 | 23590939 | F-type ATPase (Bacteria) beta |
| LOC_Os10g21266 | Chr10 | 10858911 | 10860407 | F-type ATPase (Bacteria) beta |
| LOC_Os06g39756 | Chr6 | 23590936 | 23591349 | F-type ATPase (Eukaryotes) delta |
| LOC_Os10g21264 | Chr10 | 10858501 | 10858914 | F-type ATPase (Eukaryotes) delta |
| LOC_Os10g21240 | Chr10 | 10841359 | 10842882 | V-type ATPase (Eukaryotes) B |
| LOC_Os10g21230 | Chr10 | 10839098 | 10839811 | V-type ATPase (Eukaryotes) c |
| LOC_Os01g21900 | Chr1 | 12291788 | 12294943 | inorganic pyrophosphatase [EC:3.6.1.1] |

Protein name is from the figure 5 in the manuscript.

**Table S4. Eighteen DXWR-lost transcripts during domestication**

| **Transcript ID** | **Description** | **oxidative  phosphorylation** |
| --- | --- | --- |
| c2288_g1_i1 | pf11655 family protein |  |
| c2307_g1_i1 | 40s ribosomal protein s19 |  |
| c6980_g2_i1 | non-catalytic module family expn protein |  |
| c9059_g1_i1 | hypothetical protein OsJ_13166 |  |
| c24800_g1_i3 | conserved hypothetical protein |  |
| c28691_g1_i2 | actin-related protein 9-like |  |
| c32970_g1_i1 | hypothetical protein OsI_15402 |  |
| c37812_g1_i1 | 60s ribosomal protein l6 |  |
| c39095_g1_i1 | wall-associated kinase |  |
| c44693_g1_i1 | hypothetical protein SDRG_13708 |  |
| c47871_g1_i1 | 40s ribosomal protein s5 |  |
| c48328_g1_i1 | bys1 domain |  |
| c49898_g1_i1 | nad-dependent aldehyde dehydrogenase | * |
| c52123_g1_i1 | hypothetical protein OsI_02247 |  |
| c57254_g1_i1 | cytochrome oxidase subunit i | * |
| c57519_g1_i1 | family protein |  |
| c59376_g1_i1 | papain family cysteine protease containing protein | |
| c61377_g1_i1 | cytochrome b | * |

Here, 206 transcripts were detected by comparison between the DXWR transcriptome with the Nipponbare transcriptome, which had been lost in the Dongxiang wild rice (DXWR) during its domestication. Among these 206 transcripts, 18 transcripts were annotated by the NCBI NR database. Three genes marked with * were enriched in the oxidative phosphorylation pathway.

**The parameters for bwa, SVDetect and SVFilter in command lines**

| cd /home/gaoshan/genomes  cd ./bin  path=`pwd`;export PATH="$path:$PATH"  cd ..  ./bin/bwa index -p ./databases/sativa-22 ./databases/sativa-22.fa  ./bin/bwa aln -n 2 -o 1 -e 1 -i 0 -l 50 -k 1 -t 16 ./databases/sativa-22 DNA1_1.fastq > DNA1_1.sai 2>> run.log  ./bin/bwa aln -n 2 -o 1 -e 1 -i 0 -l 50 -k 1 -t 16 ./databases/sativa-22 DNA1_2.fastq > DNA1_2.sai 2>> run.log  ./bin/bwa sampe -a 700 -f DY.pre.sam ./databases/sativa-22 DNA1_1.sai DNA1_2.sai DNA1_1.fastq DNA1_2.fastq 1>> run.log 2>> run.log  rm DNA1_1.sai  rm DNA1_2.sai  ./bin/BAM_preprocessingPairs.pl -t 1 -p 1 -d DY.pre.sam 1>> run.log 2>> run.log  rm DY.pre.sam  perl tools/pairSAMfilter.pl DY.pre.ab.sam DY.ab.sam DY.ab.sam.removed  perl tools/singleSAMfilter.pl DY.pre.norm.sam DY.norm.sam DY.norm.sam.removed  rm DY.pre.ab.sam  rm DY.pre.norm.sam  ./tools/getLength.pl databases/sativa-22.fa sativa-22.len  ./bin/SVDetect linking filtering -conf DY.sv.conf 1>> run.log 2>> run.log  perl ./tools/SVDetect2SVFilter.pl ./SVDetect_Results/DY.ab.sam.all.links.filtered DY_SV  #sed '/^@/d' .sam > result.sam  ./bin/ratiofilter DY_SV DY.norm.sam 0.2 90 1>> run.log 2>> run.log  mv DY_SV DY_SV_0  cp DY_SV_ratiofilter_kept DY_SV  ./bin/gapfilter DY_SV ./databases/sativa-22.fa 10 0.1 1>> run.log 2>> run.log  cp DY_SV_gapfilter_kept DY_SV  ./bin/SNVfilter DY_SV ./databases/sativa-22.fa DY.ab.sam DY.norm.sam 5 1>> run.log 2>> run.log  cp DY_SV_SNVfilter_kept DY_SV  samtools view -bS DY.norm.sam > DY.norm.bam  samtools sort DY.norm.bam DY.norm.sorted  samtools mpileup -f ./databases/sativa-22.fa DY.norm.sorted.bam > DY.norm.pileup  ./bin/coveragefilter DY_SV ./databases/sativa-22.fa DY.norm.pileup 6 0.1 2 1>> run.log 2>> run.log  cp DY_SV_coveragefilter_kept DY_SV  cut -f2,3 ./sativa-22.len > length.len  ./bin/depthfilter DY_SV length.len DY.norm.pileup 1.5 1.5 1>> run.log 2>> run.log  rm DY_SV  mv DY_SV_* SVDetect_Results/  mv DY.* ./SVDetect_Results/  echo "All finished!" |
| --- |
